# Effects of thickness reduction in cold rolling process on the formability of sheet metals using ANFIS

**DOI:** 10.1038/s41598-022-13694-0

**Published:** 2022-06-21

**Authors:** Yichen Xie, Yuping Wu, Arman Jalali, Huajie Zhou, Mohamed Amine Khadimallah

**Affiliations:** 1 College of Humanities and Education, Xijing University, Xi’an, 710123 Shaanxi People’s Republic of China; 2 Shanghai Jianqiao University, Shanghai, 201306 People’s Republic of China; 3 Faculty of Agriculture, Biosystems Engineering Department, University of Tabriz, Tabriz, Iran; 4 School of Engineering, University of Science and Technology Beijing, Beijing, 100083 People’s Republic of China; 5 College of Engineering, Civil Engineering Department, Prince Sattam Bin Abdulaziz University, Al-Kharj, 16273 Saudi Arabia; 6 Laboratory of Systems and Applied Mechanics, Polytechnic School of Tunisia, University of Carthage, Tunis, Tunisia

**Keywords:** Mechanical engineering, Materials for devices

## Abstract

Cold rolling has detrimental effect on the formability of sheet metals. It is, however, inevitable in producing sheet high quality surfaces. The effects of cold rolling on the forming limits of stretch sheets are not investigated comprehensively in the literature. In this study, a through experimental study is conducted to observe the effect of different cold rolling thickness reduction on the formability of sheet metals. Since the experimental procedure of such tests are costly, an artificial intelligence is also adopted to predict effects of cold thickness reduction on the formability of the sheet metals. In this regard, St14 sheets are examined using tensile, metallography, cold rolling and Nakazima’s hemi-sphere punch experiments. The obtained data are further utilized to train and test an adaptive neural network fuzzy inference system (ANFIS) model. The results indicate that cold rolling reduces the formability of the sheet metals under stretch loading condition. Moreover, the tensile behavior of the sheet alters considerably due to cold thickness reduction of the same sheet metal. The trained ANFIS model also successfully trained and tested in prediction of forming limits diagrams. This model could be used to determine forming limit strains in other thickness reduction conditions. It is discussed that determination of forming limit diagrams is not an intrinsic property of a chemical composition of the sheet metals and many other factors must be taken into account.

## Introduction

Determination of the forming limit of sheet metal is vital in designing final sheet products geometry. It is also one of the main quality control tests in deep drawing and sheet forming factories. In contrast to tensile tests, forming limit determination involves higher numbers of specimen and complicated testing procedure. In attempts to avoid such costly and time consuming tests, there are numerous analytical methods proposed in literature on the calculating FLDs using uniaxial tensile testing curve data^1–5^. However, uniaxial tensile curves cannot be a reliable source in determination of forming limits^6^. As Wu et al.^7^ experimentally showed that sheets with minor differences in uniaxial tensile curves had considerable differences in their FLDs due to the effects of their textures. Therefore, although tensile curves give intuitive sense on forming limits, it is not enough to calculate exact FLDs.

It is currently accepted that the best way to obtain the forming limit curves is experimental tests for every batch of sheet products. The forming limits are dependent on many factors including loading condition, thickness of the sheet as well as microstructural properties of the sheet. There is no model considering all geometrical, loading and microstructural parameters into account. Thus, the models cannot be reliable even they present acceptable results in specific circumstances.

Recently, there has been a rising utilization of artificial intelligence and fuzzy logic in many research fields. The effects of geometrical parameters on the forming limits were examined by Elangovan et al.^8^ using artificial neural network (ANN). The ANN model was trained using date set obtained from experiments. The trained model was further engaged to predict FLD for a set of new geometrical parameters of the sheet. The behavior of forming limit in different loading and temperature conditions were investigated by Kotkunde et al.^9^ using ANN. The predicted forming limits using ANN method were in acceptable agreement with experimental outcomes. Extreme thermal and loading rate condition are experimental difficult to apply and examine on the forming limits. In a study by Mohamed et al.^10^, it is shown that ANN could be employed to predict FLD. Derogar and Djavanroodi^11^ demonstrated the capability of ANN in sheet metal forming limit predictions.

In another trend in artificial intelligence utilization, Adaptive-Neural-network Fuzzy Inference System (ANFIS) are used to explore the problems involving fuzzy parameters^12^. This method has been become popular recently in physical modeling. For an account in forming process application, effects of geometrical parameters on the final shape of the part in hole expansion process are studied by Lu et al.^13^ using ANFIS. The initial dataset for training the ANFIS are gathered using finite element simulations of hole expansion process. The obtained results from trained ANFIS were compared to experimental outcomes. Genetic algorithm and ANFIS models were utilized by Esfahani et al.^14^ for predicting deformation in laser beam forming. ANFIS manifests remarkable efficiency in achieving accurate results with minor computational costs. There are several instances in literature using ANFIS in evaluations of material characteristics^13,15^.

Effects of different parameters on the FLD have been investigated in theoretical and experimental studies. However, in most research studies only one or two affecting parameters are considered. Influences of geometries of blank and die^16–18^, strain loading path^19^, rate of loading^20^, and lubrication^21^ were examined as mechanical parameters affecting FLD. On the other hand, second phase particles shape and distribution ^22^, production history and process^23,24^, grain size^4^, material defects^25^, texture^26–28^ and chemical composition^29^, are the some metallurgical parameters investigated forming limits determination. In simulations and theoretical modeling, neglecting effect of one parameters might result in considerable error in the FLD calculations.

In recent years, effects of microstructure of sheet material on the forming limit and workability behaviors have been reported. The effect of grain size on the formability of 316L sheets are investigated by Amelirad and Assempour^4^ using semi-real grain shapes. The simulations was conducted employing crystal plasticity theory^30^. Xu et al.^31^ experimentally examined effect of thickness to grain size ratio on the forming limits and they presented left side of the FLD. It is shown that high values of thickness to grain size ratio had detrimental effect on formability. In another study by Yamaguchi and Mellor, rise in thickness to grain size reduced limit formability of sheets^32^. Grain refinement in AZ31 is shown to improve ductility. However, formability of AZ31 sheets were independent of grain sizes as demonstrated experimentally by Azghandi et al.^33^.

Texture effects are extensively examined both experimentally and using crystal plasticity simulations. Wu et al.^7^ utilized experimental and crystal plasticity approaches to determine forming limit of aluminum sheets. They concluded that even with small difference in tensile behaviors of sheets, difference in textures leads to considerable differences in forming limit curves. Thus, the tensile curve is not a reliable source to predict forming limits without considering other factors such as texture. Barlat^28^ utilized homogenization methods of polycrystalline aggregate to obtain yield surface of sheet metal. Further, the obtained yield surface was employed to predict forming limits. Barnwal et al.^34^ demonstrated that forming limits of aluminum sheets were significantly dependent on the texture of the sheets.

To the best of author’s knowledge, influence of cold working on the formability of sheet metals has not been discussed in literature. Therefore, in this study, the application of ANFIS in determining forming limit diagrams using experimental cold rolling data is for St14 steel sheets. Experimental tensile and FLD tests are used to train and test ANFIS models. Afterwards, the trained ANFIS model is employed to predict FLD in other cold rolling conditions. The novelty of this work is to provide a simple neural-fuzzy network which could be used in place of complicated and costly experiments with an acceptable error margin. To the best of author’s knowledge, this is the first time that fuzzy logic is employed to interpret and categorize the input data for prediction of FLD.

## Experimental setup

### Heat treatment

St14 low carbon steel sheet are extensively used in forming of automobile bodies due to their high formability. A $1.5 \pm 0.03{\text{mm}}$ thickness sheet is used in this study to examined formability under different rolling conditions. The chemical composition of the sheet is presented in Table 1. The received sheet were heat treated to alleviate effects of history processes on the sheet. The heat treatment were conducted in 900 °C temperature and sheets were held for 1 h in this temperature. The sheets were cooled to room temperature in furnace. This process results in more homogenous microstructure.

**Table 1:** Chemical composition of St14 steel sheet.

C	Si	S	P	Mn	Ni	Cr	Mo	V	Ti	Cu	W	Fe
0.034	0.031	0.029	0.025	0.214	0.067	0.041	0.016	0.006	0.001	0.050	0.041	Rem.

### Cold rolling

After heat treatment, the sheets were cut into 12 cm width strips and pickled with chloride acid solution to remove oxide scales on the surface. The sheets further cold rolled to 25% and 45% thickness reduction.

### Metallography test

**Figure 1:**
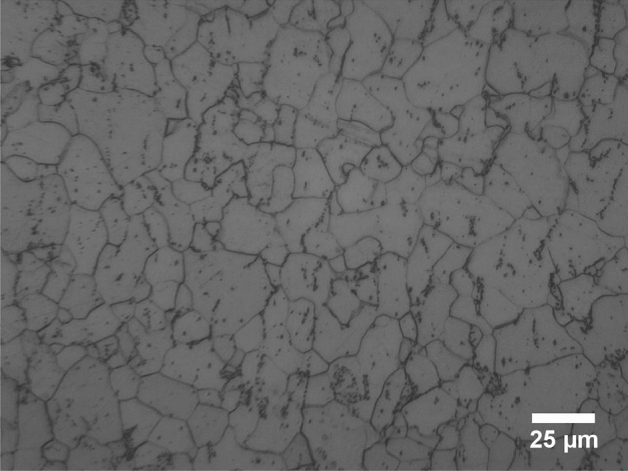
Microstructure of St14 sheet in as received condition (100×, Nital 2% for 20 s).

**Figure 2:**
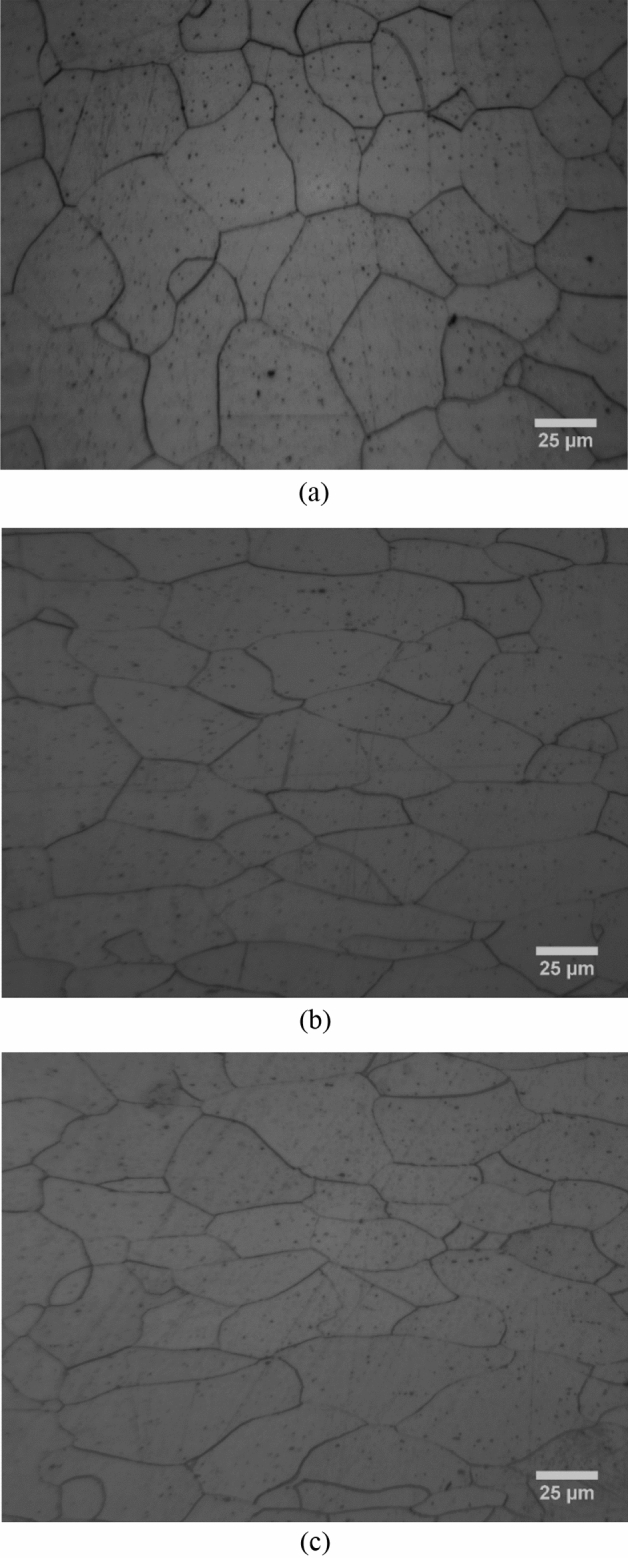
Microstructure of St14 after heat treatment (top), 25% (middle) and 45% (bottom) thickness reduction (100×, Nital 2% for 20 s).

Revealing of the microstructure of St14 steel is conducted using metallography procedure. The received specimen, heat treated and rolled ones were cut in rolling direction and sections of the sheets were mounted. Subsequently, the samples were ground using sand paper up to 3000 grit and polished with 1 μm diamond paste. Nital 2% were used to reveal grains in the sample. The results are shown in Fig. 1 for as received samples and in Fig. 2 for treated samples. As observed in Fig. 1, the grain shapes are literally spherical although slight stretch is notice in the overall structure indicating skin pass rolling after spheroidizing process. Thus the microstructure of the as received sheet metal contain spheroidized cementite. Other form of cementite would affect the structure of the sheet metal after heat treatment. After heat treatment the grains grew in size as can be seen in Fig. 2a. The grains have spherical shapes and the trace of spheroidized cementite is not seen after heat treatment indicating extensive decarburization in oxygen atmosphere of the furnace. The cold rolled microstructure is also presented in Fig. 2 for both 25% (Fig. 2b) and 45% (Fig. 2c) thickness reductions. The aim for presenting details of microstructure is its considerable effect on the forming limits such that similar thickness and chemical composition with different grain morphology results in totally different formability. For future comparisons, we believe that it is necessary to report mechanical and metallurgical properties of the sheet and testing condition as far as possible.

The average grain size in measured to be 1 μm in heat-treated and cold rolled samples.

### Tensile tests

**Figure 3:**
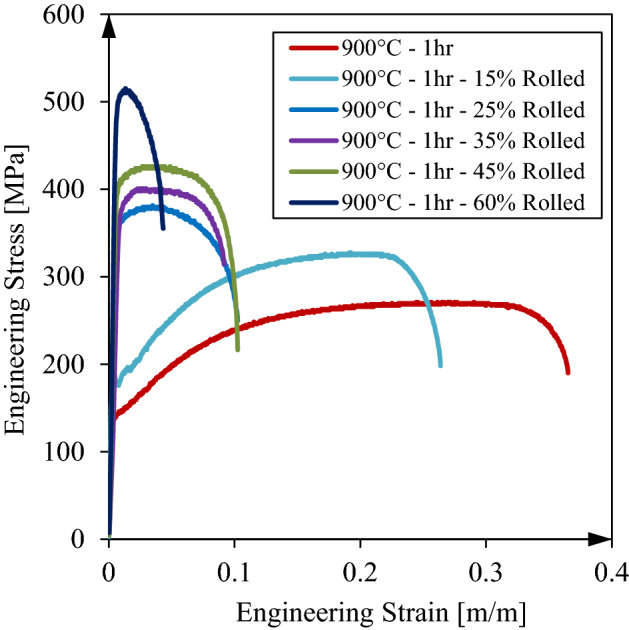
St14 uniaxial tensile curves for heat treated samples befor and after cold rolling process.

Tensile testing were conducted according to ASTM E8 standard procedure using sub-size specimens. The engineering stress–strain curves for heat treated samples are shown in Fig. 3. As seen, cold rolling significantly reduces ductility of the sheet and increase yield and ultimate strengths. Due to aging after heat treatment a slight yield point phenomena is seen the sample before rolling. The ductility reduces from 36% strain to 9.5% at rolled samples. Moreover, ultimate strength increases from 256 to 379 MPa and 438 MPa after 25% and 45% thickness reduction in cold rolling, respectively.

### Forming limit determination

**Figure 4:**
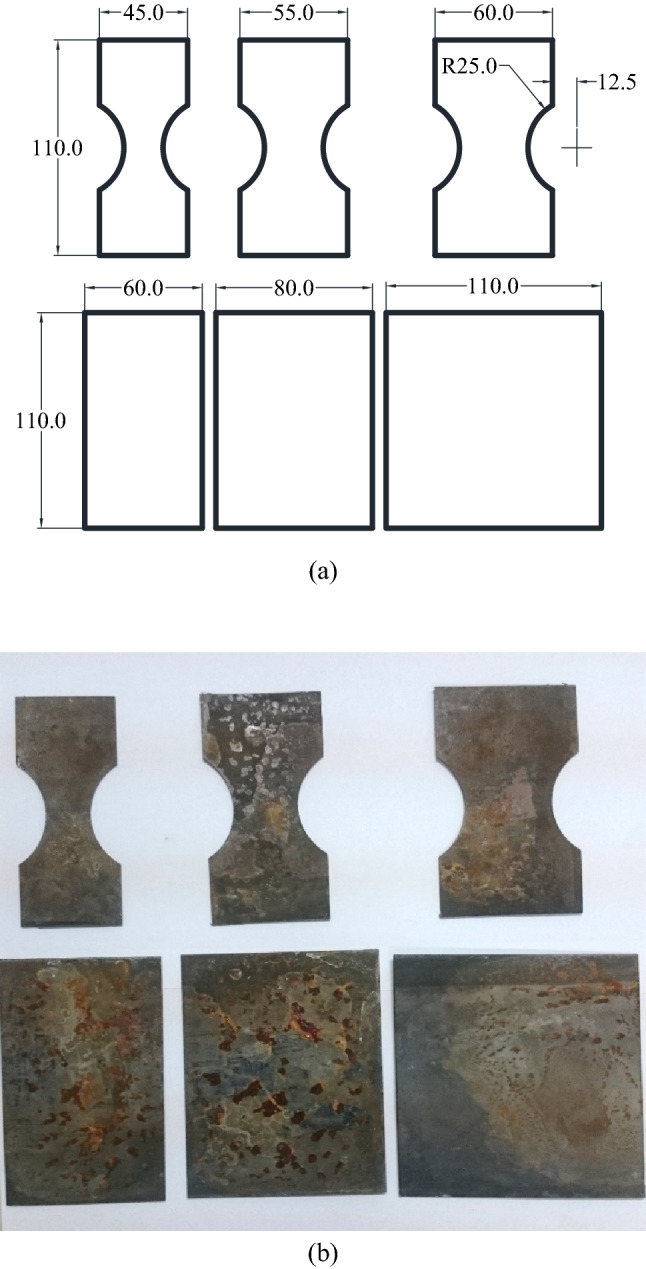
Nakazima’s forming limit samples dimensions and cut sheets. The oxide scale on the sheets were due to oxygen containing atomosphere of the furnace which was later grounded before conducting Nakazima tests.

**Figure 5:**
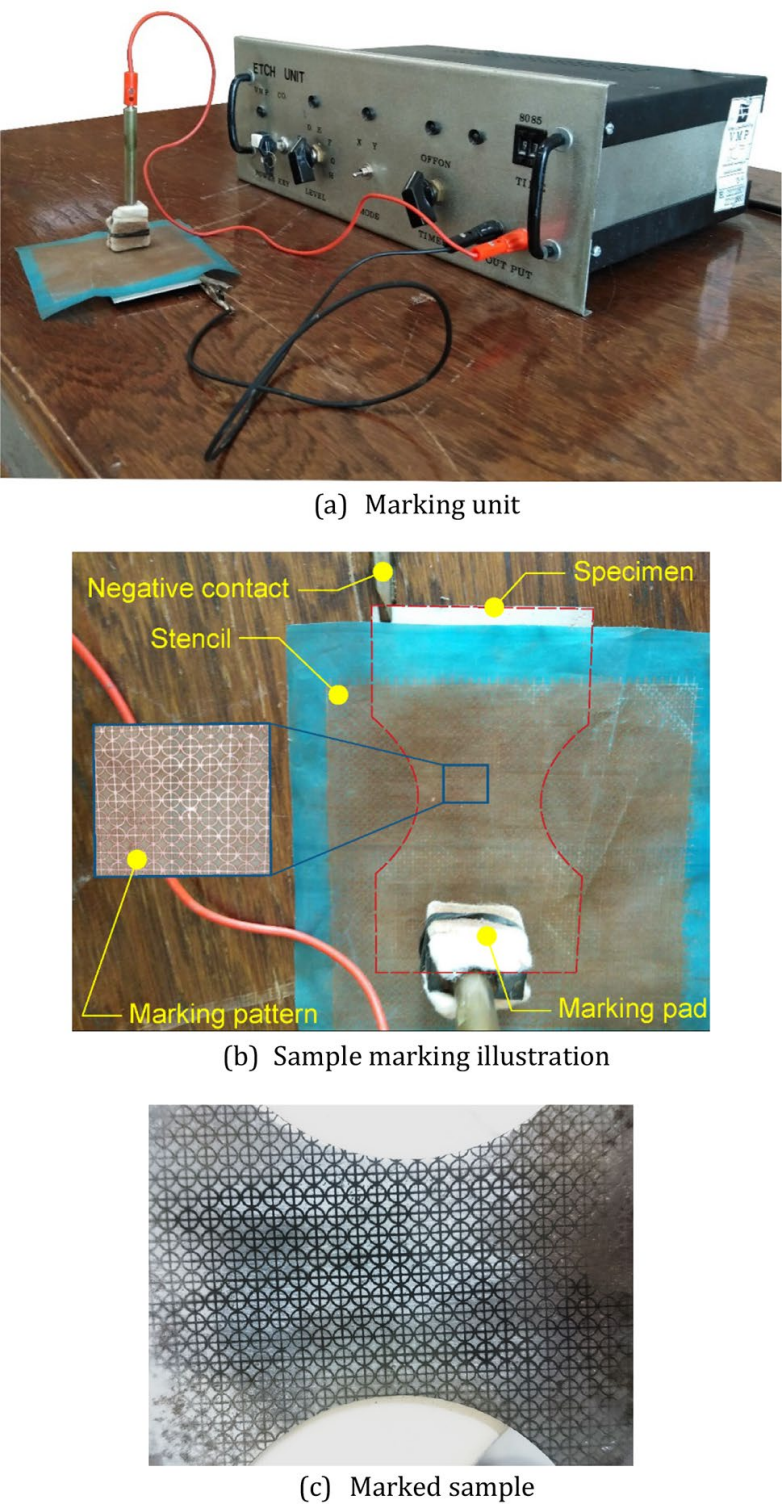
Apparatus to print circular grids on FLD samples.

Nakazima’s hemi-sphere test is utilized to determine forming limit diagrams. The sphere punch was 50 mm in diameter and all the surfaces of the punch and sheet samples were adequately lubricated to minimize friction forces. Samples were cut into bone and rectangular shapes as indicated in Fig. 4. Surface of the samples were grinded to be prepared for etch marking process. Circular and square grid marks were etched on the samples before testing to measure the major and minor principal strains after forming using apparatus shown in Fig. 5. The strains were measured using Myler strip after forming.

**Figure 6:**
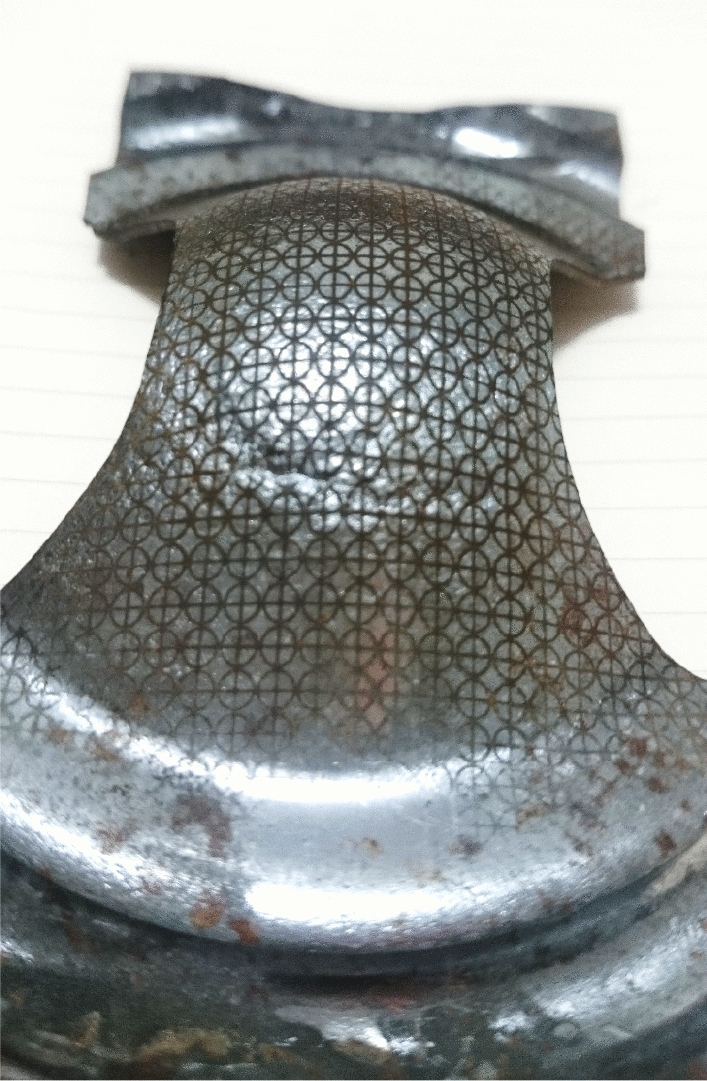
Forming limit sheet samples after Nakazima’s punch test.

**Figure 7:**
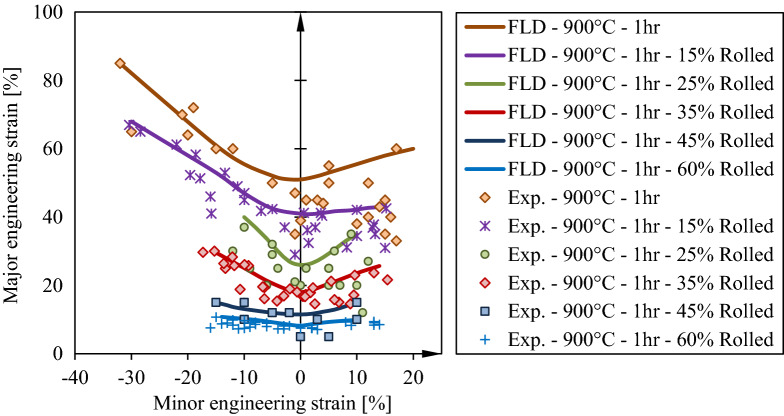
Foming limit curves of St14 steel sheets before and after cold rolling.

An example of deformed sample is presented in Fig. 6. At the region around necking and in the necking area the strains of safe and necked point are measured. Subsequently, a forming limit diagram in created using safe points of all deformed samples which represented different loading paths from unidirectional to equal bidirectional stretches. The result of FLD is shown in Fig. 7. Similar to ductility reduction, formability of the sample were significantly decreased in cold rolled sheets, and with increase in thickness reduction the forming limits reaches lower points in the diagram. This in contrast to ductility where increase in thickness reduction have negligible influence on the fracture strain for the experiments conducted in this study.

## Neuro-fuzzy system in predicting FLD method

**Figure 8:**
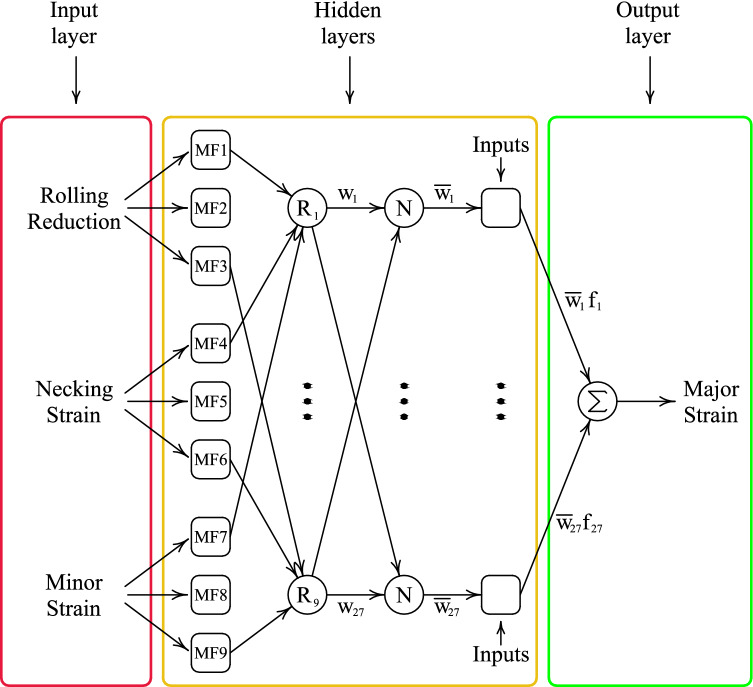
Schematic of ANFIS layers for three inputs and one outputs.

Effect of thickness reduction on the St14 sheet metals is modeled using a designed ANFIS network as depicted in Fig. 8. The advantage of using ANFIS is the use of membership functions to categorize input values. For instance, the rolling reduction can be categorized as “extreme” reduction, “moderate” reduction and “skin pass”. On this basis, different responses could be assigned to each category. In ANN, the range of all input values similar to thickness reduction values has to be fitted with the same functions. The input layer consists of three independent parameters of amount of rolling reduction, the value of strain in uniaxial tensile test at necking point and minor strain. Thus, the network has only one output major strain which will be coupled with respective minor strain to indicate a single on the forming limit curve. In the hidden layers, the first layer is called fuzzification layer in which the input values converted to their membership functions values. In total, there is maximum $3 \times 3 \times 3 = 27$ membership functions:

1 $${\text{Membership functions}} = {\text{MF}}_{i} ,{ }i = 1,2, \ldots ,27$$

The input data are assumed to have three categories each.

**Figure 9:**
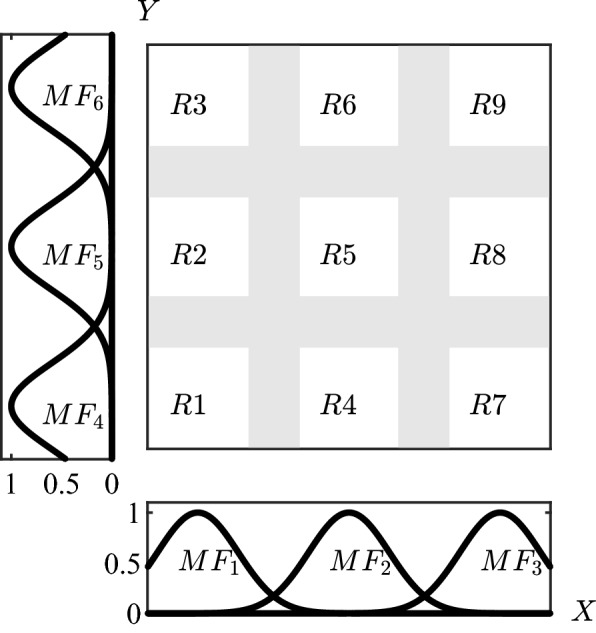
Fuzzy subspaces for ANFIS with two-inputs and three MFs.

A schematic view of relationship between rules and membership functions are shown in Fig. 9 for two inputs with 3 membership function categories. For three inputs the graph will be in 3D space. The firing strength of each rule is the combination of the MF values determined by the MF nodes. One instance of the combination is given below:

2 $$w_{i} = {\text{MF}}_{j} \left( {{\text{RR}}} \right) \times {\text{ MF}}_{k} \left( {{\text{NS}}} \right) \times {\text{MF}}_{q} \left( {{\text{MS}}} \right)$$

where RR represents rolling reduction, NS represents necking strain and MS stands for minor strain input values. To have a reasonable effect of each firing strength on the final output the output of each rule nodes are normalized according to the subsequent relation:

3 $$\overline{w}_{j} = \frac{{w_{j} }}{{\mathop \sum \nolimits_{k} w_{k} }}$$

for $$j,k = 1{\text{ to }}27$$.

In the next layer, weighted effects of the real values of the input data is calculated using Takagi and Sugeno’s relation^12,35^. The effects of the input data is considered to be linear combination of the input values:

4 $$f_{j} = p_{j} \left( {{\text{RR}}} \right) + q_{j} \left( {{\text{NS}}} \right) + r_{j} \left( {{\text{MS}}} \right) + s_{j}$$

Finally, the output is calculated using normalized firing strength and result of the Eq. (4) as given below:

5 $${{Major\,strain}} = \mathop \sum \limits_{k = 1}^{{{\text{NR}}}} \overline{w}_{k} f_{k}$$

in which $\mathrm{NR}$ represents number of all rules. This is a straight forward calculation of the input. Similar to the tuning neural networks weights and biases, adjusting membership function constants and Takagi and Sugeno’s relation is adjusted using optimization methods in several epochs. When the error margin satisfied the network is tested using the test split of the data. Caution should be exercised to avoid overfitting the data.

## Results

The input data in the ANFIS network described above is the minor strain, strain at necking and thickness reduction of the sheets. The dataset is prepared based on the experimental results of tensile testing and FLD determination. As a rule of thumb for any prediction made by the network there has to be at least 10 times data in the network. In our case, we are aiming to predict one FLD curve which contains at least 5 points (two points on the right side, two points on the left side and one FLD0 point). Therefore, at least 50 sets of data is required to train the ANFIS modelThe input and output data are given in Table 2. Triangle membership functions are considered for fuzzification of the input data:

6 $$f\left( {x;a,b,c} \right) = \max \left( {min \left( {\frac{x - a}{{b - a}},\frac{c - x}{{c - b}}} \right),0} \right)$$

**Table 2:** Input and output data based on experimental tests.

Run	Thickness reduction (%)	Necking strain (%)	$\epsilon_{2}$(%)	$\epsilon_{1}$[%]
1	0	27	− 32	85
2	0	27	− 25	75
3	0	27	− 15	61
4	0	27	− 10	55.5
5	0	27	− 5	52.1
6	0	27	− 3	51.2
7	0	27	0	51
8	0	27	3	52
9	0	27	5	53
10	0	27	10	55.5
11	0	27	15	58
12	0	27	20	60
13	15	18	− 30	68
14	15	18	− 25	63
15	15	18	− 15	53
16	15	18	− 10	47
17	15	18	− 5	42.5
18	15	18	0	41
19	15	18	3	41
20	15	18	6	41.7
21	15	18	9	42
22	15	18	12	42.7
23	15	18	15	43
24	25	5	− 10	40
25	25	5	− 8	37
26	25	5	− 6	33.7
27	25	5	− 4	30
28	25	5	− 2	27
29	25	5	0	26
30	25	5	2	26.5
31	25	5	4	28.2
32	25	5	6	30.5
33	25	5	8	33
34	25	5	10	35
35	45	4	− 15	15
36	45	4	− 13	14.2
37	45	4	− 12	13.7
38	45	4	− 10	13.1
39	45	4	− 5	12
40	45	4	− 2	11.6
41	45	4	0	11.5
42	45	4	2	11.7
43	45	4	4	12.3
44	45	4	6	12.9
45	45	4	8	13.9
46	45	4	10	15
47	60	2.4	− 14	10.8
48	60	2.4	− 12	10.5
49	60	2.4	− 8	9.9
50	60	2.4	− 4	9
51	60	2.4	− 1	8.3
52	60	2.4	0	8.2
53	60	2.4	1	8.4
54	60	2.4	3	8.9
55	60	2.4	5	9.3
56	60	2.4	8	9.7
57	60	2.4	9	9.8

The parameters $a$, $b$ and $c$ are determined assigned by ANFIS app in Matlab.

**Figure 10:**
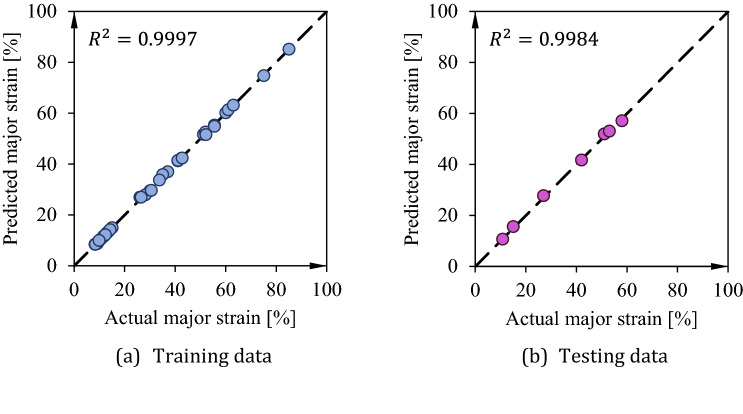
Comparison of the outputs of ANFIS and experimental values for both training and testing data.

In Table 2, 14 set of data are used to train the network and 4 remaining are testing data to ensure about reliability of the network. After iteration, the overall error is 1.9% and the predicted values for bot training and testing data is demonstrated in Fig. 10. As seen, the train network predicts results with high accuracy. The trained network surface for the thickness reduction is shown in.

**Figure 11:**
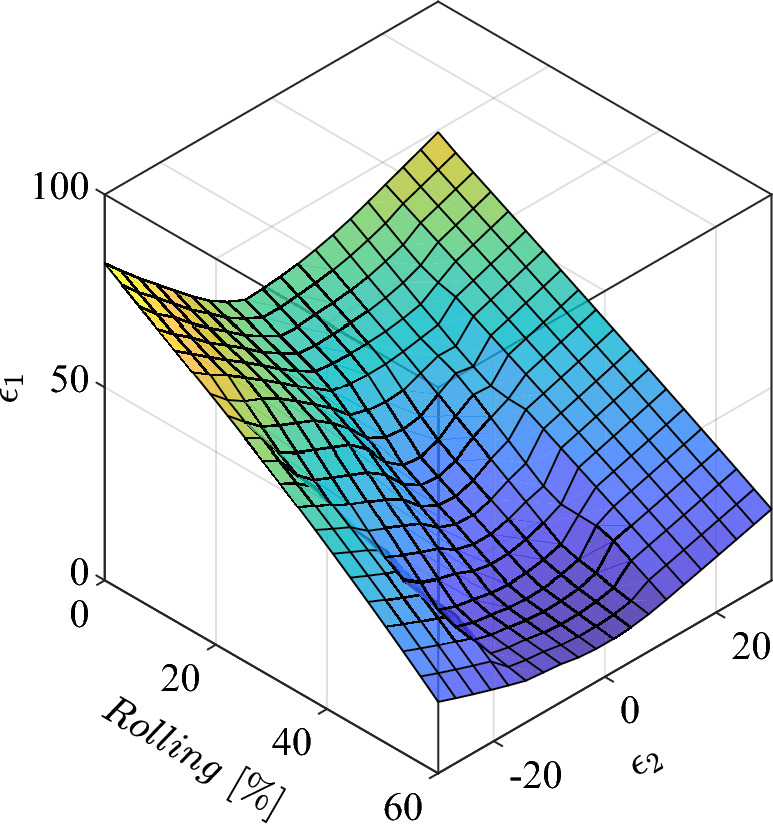
Effects of Thickness reduction in cold rolling process on forming limit curves for St14 sheet steel.

The trained network surface for the thickness reduction is shown in Fig. 11. Using this surface, it is possible to generate new FLD for any thickness reduction other than those given by experiment. Based on the accuracy of trained model, for the case of these material and processing condition, this surface is highly reliable. Similar procedure can be adopted for any other processes in industry. Because the processes in the production line are stable for many circumstances. Another benefit of such networks is simplicity and capability of adjustment using new provided data.

**Figure 12:**
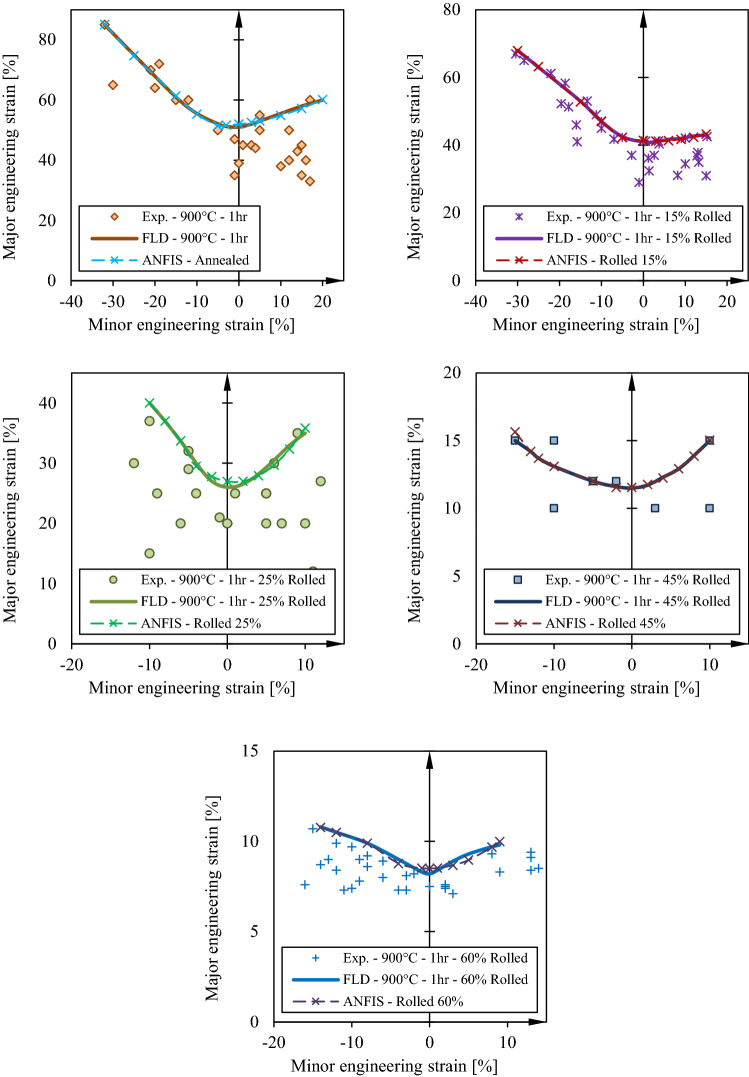
Comparison of experimental and simulation forming limits for St14 in different annealing conditions.

The ANFIS model trained and tests in the previous section is now used to predict FLD under different rolling conditions and the results are compared to the experiments. In Fig. 12, it is shown that forming limit curve generated by ANFIS model is in perfect agreement with the experimental curve. In generating these curves by ANFIS several extra point in minor strain is utilized. This predictions can be generalized to incorporate other parameters in the ANFIS model. In other methods incorporating one more parameters in the calculations results in tremendous time and computational costs while in ANFIS it can be added with minimal effort^4,18,24,36–38^.

**Figure 13:**
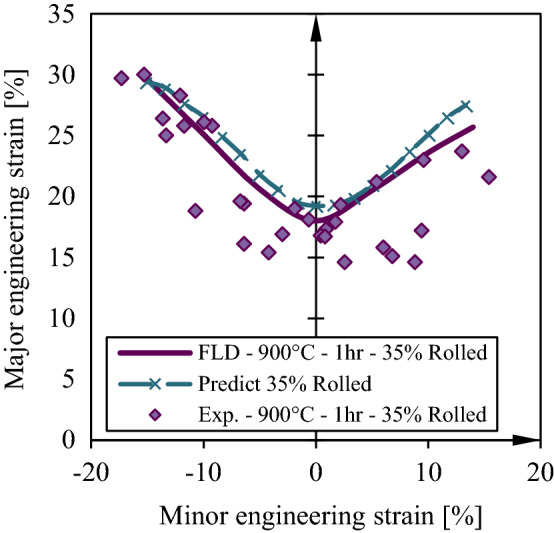
Effects of Thickness reduction in cold rolling process on forming limit curves for St14 sheet steel.

In the data selected for the purpose of training and testing ANFIS network, the data of the samples of 35% cold rolled are deliberately dropped. In this section, the trained and tested model is utilized to predict the forming limit curve of the sheet with 35% thickness reduction providing only the values of necking strain and thickness reduction to the network. The output FLD is depicted in Fig. 13 and it is compared with the experimental data and FLD. It is clear that the trained ANFIS model satisfactorily with 6.5% error which is regarded a low error in comparison to other methods of FLD prediction^4,26,39^.

## Conclusion

In this study, the detrimental effect of cold rolling on the formability of sheet metals was examined using experimental tests and neural-fuzzy model. The importance of using ANFIS model, if properly trained, was avoiding time consuming and laborious experimental tasks. To the author’s knowledge, this is the first attempt in engaging ANFIS in prediction of forming limits of sheet metals. The experimental works were conducted using St14 heat treated sheet metals. The sheets were cold rolled to different thickness reduction and tensile, metallography and FLD tests are conducted to determine forming behavior and properties of the sheets. An ANFIS model is also designed to predict effects of cold thickness reduction on the formability of the sheet metals. The obtained data from experiments were utilized to train and test the ANFIS model. The main conclusion of the results are:

- Cold rolling of sheets significantly reduces the formability of sheets. Increase in the thickness reduction results in less formability
- Experimental methods in determining FLD is costly and proposed models in the literature have not considered all mechanical and microstructural parameters influencing FLD.
- Artificial intelligence methods are low cost and light weight method that can incorporate several parameters into consideration.
- ANFIS model can include effects of cold rolling in prediction of FLD and the difference between experimental and ANFIS results are negligible.
